# Artificial Intelligence System for Predicting Prostate Cancer Lesions from Shear Wave Elastography Measurements

**DOI:** 10.3390/curroncol29060336

**Published:** 2022-06-10

**Authors:** Ciprian Cosmin Secasan, Darian Onchis, Razvan Bardan, Alin Cumpanas, Dorin Novacescu, Corina Botoca, Alis Dema, Ioan Sporea

**Affiliations:** 1 Department of Urology, “Victor Babes” University of Medicine and Pharmacy, 300041 Timisoara, Romania; cosminsecasan@yahoo.com (C.C.S.); alincumpanas@hotmail.com (A.C.); dorin.novacescu@yahoo.com (D.N.); 2 Department of Urology, “Pius Brinzeu” Clinical Emergency County Hospital, 300736 Timisoara, Romania; 3 Department of Computer Science, West University, 300223 Timisoara, Romania; darian.onchis@e-uvt.ro; 4 Department of Communications, Polytechnic University, 300006 Timisoara, Romania; corina.botoca@upt.ro; 5 Department of Pathology, “Victor Babes” University of Medicine and Pharmacy, 300041 Timisoara, Romania; dema_alis@yahoo.com; 6 Department of Gastroenterology, “Victor Babes” University of Medicine and Pharmacy, 300041 Timisoara, Romania; isporea@umft.ro

**Keywords:** artificial intelligence system, shear wave elastography, prostate cancer

## Abstract

(1) Objective: To design an artificial intelligence system for prostate cancer prediction using the data obtained by shear wave elastography of the prostate, by comparing it with the histopathological exam of the prostate biopsy specimens. (2) Material and methods: We have conducted a prospective study on 356 patients undergoing transrectal ultrasound-guided prostate biopsy, for suspicion of prostate cancer. All patients were examined using bi-dimensional shear wave ultrasonography, which was followed by standard systematic transrectal prostate biopsy. The mean elasticity of each of the twelve systematic biopsy target zones was recorded and compared with the pathological examination results in all patients. The final dataset has included data from 223 patients with confirmed prostate cancer. Three machine learning classification algorithms (logistic regression, a decision tree classifier and a dense neural network) were implemented and their performance in predicting the positive lesions from the elastographic data measurements was assessed. (3) Results: The area under the curve (AUC) results were as follows: for logistic regression—0.88, for decision tree classifier—0.78 and for the dense neural network—0.94. Further use of an upsampling strategy for the training set of the neural network slightly improved its performance. Using an ensemble learning model, which combined the three machine learning models, we have obtained a final accuracy of 98%. (4) Conclusions: Bi-dimensional shear wave elastography could be very useful in predicting prostate cancer lesions, especially when it benefits from the computational power of artificial intelligence and machine learning algorithms.

## 1. Introduction

Prostate cancer (PCa) is the most common malignancy diagnosed in Western men, and represents the second highest cause of cancer death in men [1]. Despite the recent general advances in prostate imaging and genetic testing, the actual diagnosis of PCa is still based on the dosing of total serum prostate specific antigen (PSA), the digital rectal examination (DRE) as screening methods and on the ultrasound-guided 12-core systematic prostate biopsy as confirmation method [2].

Grey-scale transrectal ultrasonography (TRUS) is used during the transrectal or transperineal prostate biopsy as the preferred mapping method of the prostatic regions, although it has relatively low sensitivity and specificity for detecting PCa lesions, as the suspicious hypoechoic areas are not confirmed during biopsy in 60–80% of the cases [2]. In order to increase the precision of prostate imaging, newer techniques including multiparametric magnetic resonance imaging (mp-MRI) have been developed and extensively tested, but they unfortunately involve longer waiting times and significant costs [3].

In the last twenty years, ultrasound-based elastography has become an interesting method for the assessment of organ stiffness [4]. More recently, elastographic targeted prostate biopsy has become an alternative for conventional ultrasound-guided systematic biopsy, as the studies of Mitterberger and Junker have shown that a 50% reduction of the number of biopsy cores using elastography offered the same prostate cancer detection rate as the standard randomized TRUS-guided biopsy [5,6,7,8].

The principle of elastography consists in its ability to differentiate the tissue structures according to their elasticity, as opposed to the standard ultrasonography, which differentiates them through acoustic impedance [9,10,11,12]. Actually, there are two elastographic methods used in medicine: strain and shear wave elastography (SWE). Shear wave elastography generates shear waves using a sonographic push pulse; the generated image expresses tissue stiffness in a color-coded map [13]. SWE methods include transient elastography (used for the assessment of liver stiffness), point shear wave elastography (pSWE) and bi-dimensional shear wave elastography (2D-SWE) [14]. For the evaluation of prostate lesions, 2D-SWE represents an attractive alternative, as tumor tissue is associated with an increased density of cells and vessels, which is translated as a reduction of tissue elasticity [5,15].

In recent years, medicine began to use artificial intelligence (AI) to help medical practitioners in decision making, analyzing a high number of parameters in a very short time and finally proposing solutions with a high level of confidence. AI is increasingly used in daily medical practice, processing data for more patients, with a higher level of confidence, from a myriad of systems and sensors [16].

AI is an innovative computer science discipline that aims to imitate and in some specific tasks to completely replace human thinking. Following the development in the fields of information technologies and computational power, several types of advanced early detection models were developed, enabling the computer systems to learn patterns from labeled data, in order to classify or to predict an output with a high statistical confidence [17,18]. These models include, along with logistic regression (extensively used in the previous decades), newer techniques as decision trees, support vector machines and artificial neural networks [19,20,21].

Machine learning is a subset of AI which is capable of learning from experience without being previously programmed to produce specific outputs, while deep learning is a subset of machine learning, with a structure very similar to the physiology of the human brain, able to use large datasets at the same time, which are reprocessed multiple times, in order to reach a desired output [22]. Thus, deep learning is a very innovative tool, using multi-layer neural networks to develop high-precision predictive models [18,23]. Deep learning algorithms usually succeed in obtaining better accuracy and receiver operating characteristic curves compared with more conventional analysis tools applied to the same dataset [24].

In urology, deep learning algorithms were successfully used for the detection of prostate cancer, correlating mp-MRI images with the results of prostate biopsy, and for the outcome prediction after the robot-assisted radical prostatectomy, delivering an average performance increase of 30–80%, compared with traditional diagnostic standards [25,26]. Moreover, machine learning algorithms provide, besides state of the art solutions, the much needed continuous adaptation and improvement through learning, and therefore were the obvious choice for completing the algorithmic part of our intelligent prediction system.

## 2. Aim of the Study

Our main objective was to design an artificial intelligence system able to predict prostate cancer using the data obtained by shear wave elastography of the prostate, by comparing it with the data gathered by the actual diagnosis gold standard, the histopathological exam of the prostate biopsy specimens.

## 3. Material and Methods

We have performed a prospective study, which included patients from our outpatient department, scheduled for transrectal ultrasound-guided prostate biopsy, for prostate cancer suspicion, from January 2017 to November 2019. The study was approved by the local Ethics Committee of our Hospital, and all participating patients have signed an informed consent form.

We have used the following inclusion criteria for patient selection: at least one total Prostate Specific Antigen (PSA) value over 4 ng/mL and/or an abnormal digital rectal examination, raising the suspicion of prostate cancer. Subsequently, we have applied the following exclusion criteria: past history of prostate cancer, past history of surgical/endoscopic procedures of the lower urinary tract, recent history or signs of acute/subacute prostatitis.

The following demographic and clinical parameters were recorded in all patients: age, PSA value and interpretation of digital rectal examination performed by the same urologist. In some of the patients, we gathered additional data, including free PSA, body mass index (BMI), International Prostate Symptom Score (IPSS) value and the interpretation of multiparametric prostate magnetic resonance imaging (mp-MRI). Since we were not able to provide these parameters in all patients, we preferred to not include them in our actual study, which evaluates the patient group with homogenous.

All patients have received a prophylactic dose of Levofloxacin 500 mg, 24 h before the biopsy and another dose of 500 mg in the morning of the procedure. Some of the patients were instructed to continue antibiotic therapy if they were considered at risk for post-biopsy infection.

The combined approach, including conventional transrectal ultrasonography and shear-wave elastography, was performed with a Aixplorer Supersonic Imagine machine (Aixplorer, Aix-en-Provence, France), using a SE12-3 transrectal transducer, which had a Civco needle guide system attached (Civco, Coralville, IA, USA), for directly performing the biopsy right after the elastographic image acquisition.

**Figure 1:**
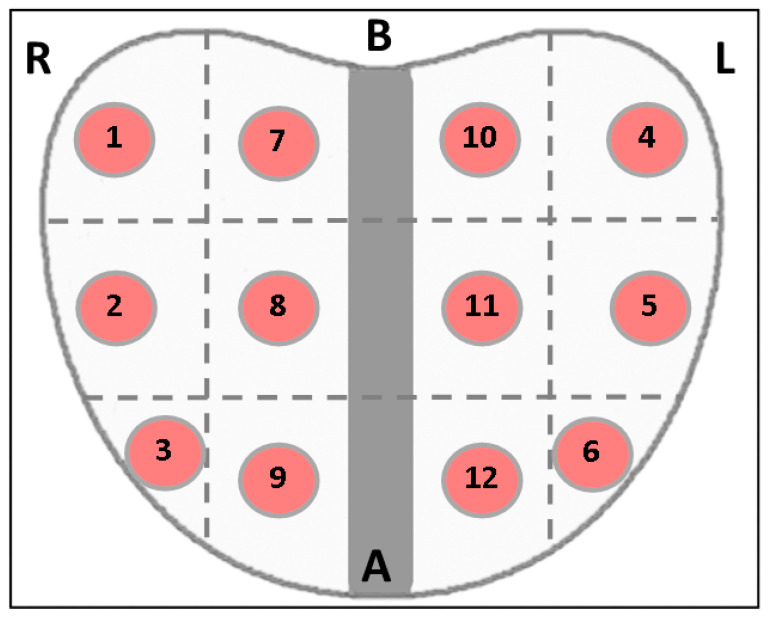
Prostate divided into twelve target zones, resulting a total of twelve biopsy fragments at approximatively 1 cm distance between each other. Every target zone and tissue fragment corresponds to a region evaluated with SWE measurements before the biopsies were taken (R = right, L = left, B = base and A = apex).

After the insertion of the transrectal transducer with the patient in lithotomy position, we have started our ultrasonographic evaluation. The prostate was divided into twelve circular target zones with a diameter of 5 mm, six peripheral and six para-urethral, at approximately 1 cm distance between each other, as shown in Figure 1. Each area was evaluated initially for the existence of hypoechoic lesions in gray-scale ultrasonography, which was followed by bidimensional shear wave elastography.

### 3.1. Methodology for Elastography

**Figure 2:**
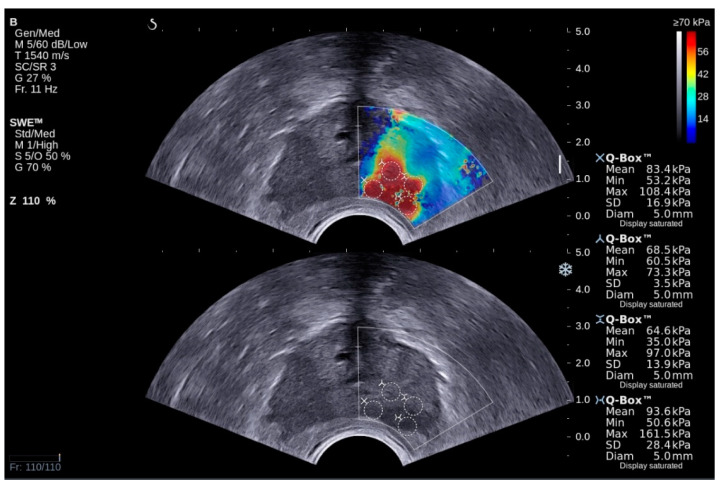
Concomitant transrectal visualization of the prostate bidimensional shear wave elastography (upper image) and grey-scale ultrasonography (lower image). Four regions of interest raise the suspicion of PCa (colored in red) at the level of the left prostatic lobe, and three of them were targeted during transrectal prostate biopsy. The pathology report has confirmed the PCa lesions in all three biopsy cores.

Bidimensional shear wave elastography (2D-SWE) was utilized to visualize and measure the rigidity of the target zones considered for biopsy. The rigid regions appeared colored in red, while those soft and elastic were colored in blue. Hard regions with a diameter of at least 5 mm were considered suspicious and potentially malignant (Figure 2). Using the software implementation of the Young module of the ultrasound machine, we have calculated the mean elasticity of each of the target zones.

The 2D-SWE assessment of the central area around the urethra was excluded, as this area is often very stiff and difficult to evaluate. Accurate SWE results are generally not reproducible, while the probability for detecting a prostate cancer lesion is very low [7].

### 3.2. Methodology for Prostate Biopsy

**Figure 3:**
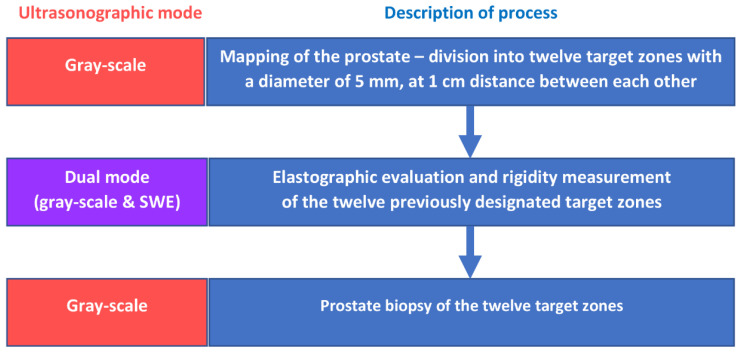
Scheme of the image processing and systematic biopsy of the twelve target areas.

Following the elastographic evaluation, we have performed local anesthesia, using 10 mL lidocaine 1%, injected through a 22 G needle. Thereafter, we have performed the transrectal prostate biopsy, from the twelve target zones, using an 18 G biopsy needle attached to a BARD Magnum biopsy instrument (Bard Care, Covington, GA, USA) (Figure 3). No additional biopsy cores were included in our analysis, even if the elastographic examination indicated a suspicious lesion outside the target zones, and we have harvested some additional fragments from those areas. The biopsy core specimens were blinded to the results of the elastographic measurements and were processed using the standard procedures of the Pathology Department from our Hospital. The pathology report was made available for each of the biopsy cores, including data about tumor linear extension and Gleason score, in confirmed PCa positive lesions.

### 3.3. Creation of the Dataset

The patients were included in the dataset only when the pathology report from the prostate biopsy was finalized and included at least one core fragment confirmed with prostate cancer; thus, all the patients with negative biopsy results, or with lesions such high grade prostatic intraepithelial neoplasia (HG-PIN) or atypical small acinar proliferation (ASAP) were excluded, as our intention was to train the AI system only with confirmed cancer cases. The dataset included the imaging parameters for each of the twelve target zones (mean elasticity, measured in kPa), and the corresponding biopsy results, marked with 0 for negative (no cancer) and with 1 for positive (confirmed cancer).

### 3.4. Implementation of Machine Learning Techniques

We have employed artificial intelligence techniques, designing a dynamical auto-adaptive system customized for analyzing our dataset. We have implemented three machine learning classification algorithms, namely the logistic regression, a decision tree classifier and a fully connected feed-forward deep neural network [27,28,29].

Logistic regression is the first algorithm to try in a classification problem, and generally has the lowest accuracy and the lowest weight in the total ensemble. Decision trees and their ensembles as random forests are good shallow classifiers. The deep neural network was chosen as our aim was to predict with the highest possible accuracy the positive diagnosis of prostate cancer using the numerical values associated to the elastographic regions of interest measured before the biopsy.

### 3.5. Neural Network Classifier Implementation

We have constructed our neural network classifier in TensorFlow version 2.5.0 (Google, Mountain View, CA, USA), using Keras, as it is one of the most commonly used Python-based deep learning platforms, both among researchers and industry, with tools for building almost any type of neural network architecture [30,31]. For the logistic regression and decision tree, we have used the Python library Scikit-Learn [27,28].

The deep learning network was optimized for both the parameters (i.e., weights) to avoid overfitting, and hyper parameters (i.e., number of layers) to increase the classification accuracy. We have used the ADAM (adaptive moment estimation) optimization algorithm instead of the classical stochastic gradient descend procedure to update network weights in an iterative way ADAM updates a learning rate separately for each model parameter/weight, increasing the learning rate in the early layers, and thus improving the efficiency of deep neural networks [32].

**Figure 4:**
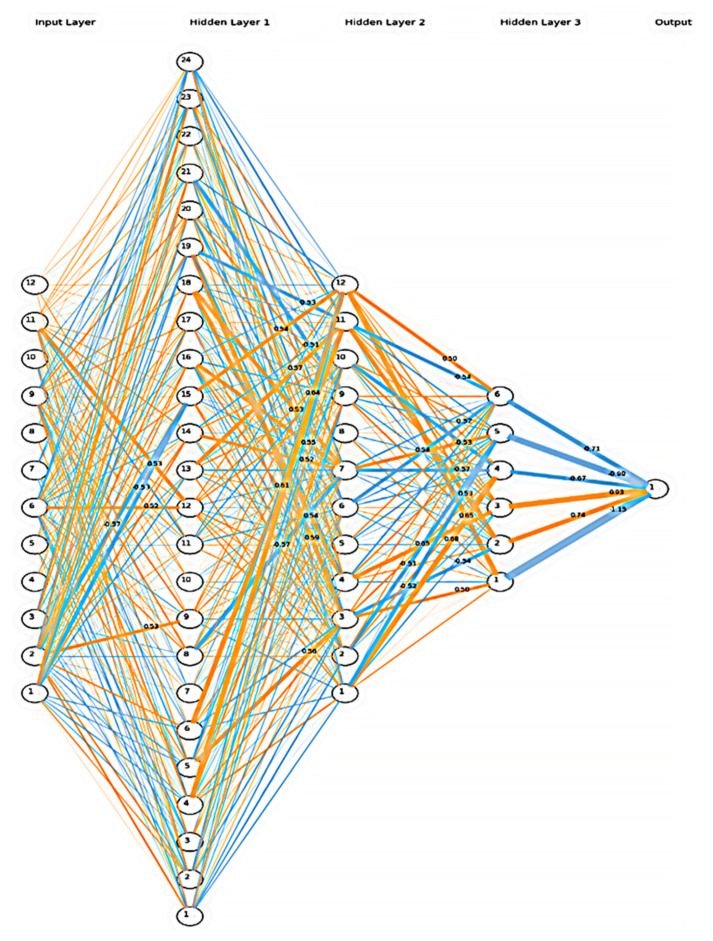
Visualization of the deep learning model. The neurons represented by the small circles are numbered from the bottom to top on each layer in ascending order. The first layer is the input layer with the elasticity values. The remaining four processing layers (three hidden and one output) are described in Table 1. Activation weights are marked with a thicker line.

We have considered finally the following model (Table 1):

The deep learning model with the activated weights after the training can be visualized in Figure 4:

### 3.6. Statistical Analysis

We have used as performance evaluation metrics the receiver operating characteristic curve (ROC) created by plotting the true positive rate (TPR) against the false positive rate (FPR), at various threshold settings [33]. We have utilized for normalized units the area under the curve (AUC), which is equal to the probability that a classifier will rank a randomly chosen positive instance higher than a randomly chosen negative instance. Moreover, we have calculated the sensitivity and specificity since they are statistical measures of the performance of a binary classification test.

Cross-validation was performed, using the k-fold procedure, which divides all the samples in groups of samples, called folds, of equal sizes. The prediction function was learned using folds, and the fold left out was used for testing.

## 4. Results

**Figure 5:**
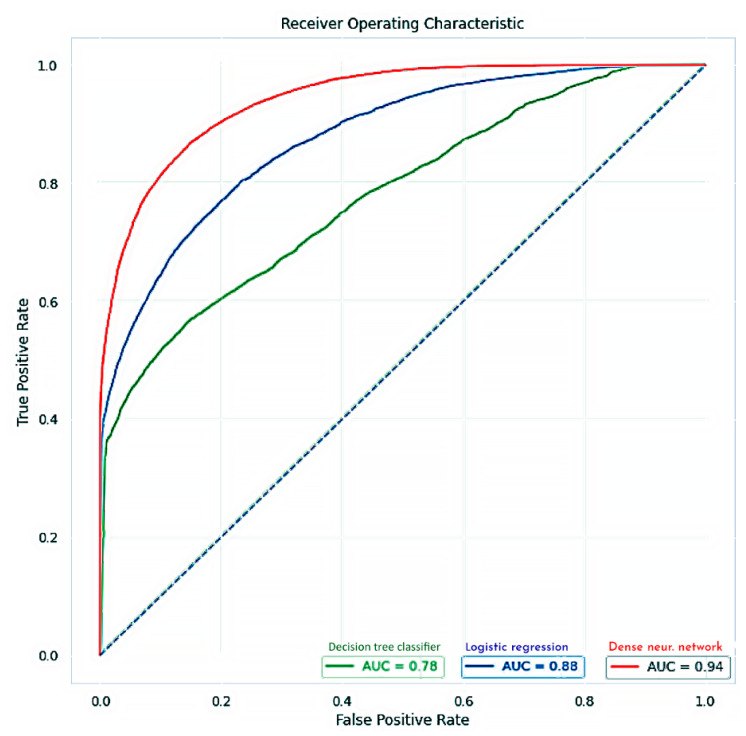
ROC curves of the three systems used for prediction: logistic regression (trained with gradient descent instead or ordinary least squares, marked with blue; AUC = 0.88), decision tree classifier (using the ID3 algorithm, marked with green; AUC = 0.78), and dense neural network (with three hidden layers, marked with red; AUC = 0.94).

From the total of 356 screened patients, which underwent the evaluations from our protocol, a number of 223 were confirmed with prostate cancer. Their mean age was 64.2 years (standard deviation 11.9 years), and the mean PSA value was 18.9 ng/mL. Using the Gleason score from the pathology reports, we have obtained the following table, which correlates the International Society of Urologic Pathologists (ISUP) grading with the average PSA and the percentage of positive cores (Table 2):

After the finalization of the dataset, using the described parameters, we have performed the three simulations, obtaining the following results (Table 3):

The receiver operating characteristics of the three systems are presented in Figure 5:

### 4.1. Re-Training of the Neural Network

Comparing our results, we have obtained the highest accuracy with the neural network classifier (AUC = 0.94), followed by the logistic regression (AUC = 0.88), and the decision tree (AUC = 0.78). The differences between the three classifiers reside, probably, from a slight imbalance between the positive and negative prostate cancer biopsy cores. In order to minimize this imbalance, we have used the upsampling strategy SMOTE (synthetic minority oversampling technique) [34]. We used it only for the training set, such as the newly generated samples will not influence the model prediction. After applying SMOTE, we have obtained a well-balanced dataset; by retraining the deep neural network classifier, the prediction was slightly improved, reaching an AUC = 0.95.

### 4.2. Ensemble Learning Model

Since we needed to further improve the accuracy of our model, we have employed a weighted ensemble learning strategy. Ensemble methods are techniques that combine the inferences of multiple machine learning models with the purpose to produce improved results [35]. Ensemble methods are usually more accurate solutions than a single model.

We have combined the results from our three models, and we have employed a majority voting and then a weighted voting, implemented using the Sci-kit learn library. In the first scenario, we have used as votes the predictions from all our three models for each test instance and the final output prediction was the one that received more than half of the votes. Since we observed that the neural network usually produces the highest accuracy prediction, we have decided to slightly upgrade the weight of its vote and we have given similar weights for the logistic regression and decision tree. Using this ensemble learning strategy, we were able to obtain a final accuracy of 98%, which is adequate given the size of the original dataset. Since the dataset is under continuous update by incorporating new patients, our future goal is to apply an incremental learning classifier (such as e.g., ICARL) to further develop our system [36].

## 5. Discussion

Our main objective was to prospectively evaluate the diagnostic performance of shear-wave elastography (SWE) in the detection of prostate cancer, by designing an intelligent system composed of a clinical part and an ensemble learning part, where we have compared the predictions of 3 supervised classifiers, a logistic regression system, a decision tree and a dense neural network.

As the sensitivity of TRUS-guided prostate biopsy in detecting PCa remains relatively low, additional imaging techniques have been evaluated. Most of the actual studies are concentrated on the role of multiparametric prostate MRI, and on the use of MRI/TRUS fusion biopsy, with significant positive results, entitling some of the authors to declare that the era of randomized systematic biopsy is starting to fade away [3]. Despite these statements, there are some elements that need to be considered. First, mp-MRI is a time-consuming procedure, which requires a high level of technician and physician training and sophisticated equipment, in order to provide good quality scans and standardized reports [37]. Second, fusion biopsy requires expensive ultrasound machines and proper training, but is sometimes not able to give reproducible results, as still depends very much on the type of fusion technology and equipment [38]. Of course, artificial intelligence could be a game changer in this field, too, recent advances incorporating the use of AI into the fusion software, with proven positive results [39].

On the other hand, the development of elastography in the last two decades has improved the non-invasive assessment measuring the organ stiffness [40]. Most of the studies were focused on liver elastography, but a lot of work was performed more recently for the evaluation of the breast, thyroid, spleen or prostate [41]. For the assessment of prostate stiffness, 2D-SWE is the most suitable method, as color coded imaging enables the detection and evaluation of stiff areas, which raise the suspicion of prostate cancer, with a higher sensitivity than the standard ultrasonography.

Several research groups have investigated the accuracy of real time elastography in correlation with biopsy results for the detection of PCa. The most important limitation of 2D-SWE is represented by its low performance in detecting tumors smaller than 5 mm [42]. Moreover, in order to determine the true sensitivity and specificity of the method, a correlation with histo-pathological sections of radical prostatectomy specimens is finally required [43]. A further limitation of the technique is represented by the operator subjectivity in detecting prostate areas with high rigidity. In this matter, it is very important to standardize the elastography method as much as possible, in order to increase the accuracy of the measurements. False positive aspects raising the suspicion for PCa may be encountered in chronic prostatitis, due to the “distance” and “attenuation” effects, in adenomatous nodules, or due to the “striated” appearance of the base, often difficult to recognize. False negative aspects, especially in the transitional zone, can be caused by any lesion that may “hide” information, as intraprostatic calcifications, or peripheral infiltrative lesions with capsule penetration [40].

Our current research has demonstrated the value of AI, which can significantly simplify the prediction procedures, streamlining the decision making, reducing the bias and errors due to methodological mistakes, and outperforming the standard statistical methods. Of course, one of its drawbacks resides right from its ability to build non-linear response functions, which are harder to explain, despite their remarkable accuracy [25].

## 6. Conclusions and Future Work

Our intelligent system finally provided a high level of accuracy, but considering the relatively moderate size of the dataset, did not allow us to omit the randomized systematic biopsy in favor of the SWE-targeted biopsy.

As a future development of our research, we intend to enhance the performance of a new combined technique, of multiparametric ultrasonography of the prostate, which uses B-mode imaging, SWE and contrast-enhanced ultrasound with parametric maps, by adding the computational and prediction capabilities of artificial intelligence systems, including model-agnostic explainable artificial intelligence techniques such as LIME (local interpretable model agnostic explanation) [44,45,46]. Moreover, future implementations of these techniques should take into account the capacity to differentiate between indolent and potentially aggressive PCa lesions, reducing the need for systematic prostate biopsies and minimizing the complication risks [47].

## Figures and Tables

**Table 1:** The structure of the dense neural network.

Layer (Type)	Output Shape	Parameter Number
dense_1 (Dense)	(None, 24)	312
dense_2 (Dense)	(None, 12)	300
dense_3 (Dense)	(None, 6)	78
dense_4 (Dense)	(None, 1)	7

**Table 2:** Correlations between the ISUP grading and different patient parameters.

	No.	Mean Age	Mean PSA	% of Positive Cores	% of DRE Positive
ISUP 1	90	63.21	10.915	34.2%	16.7%
ISUP 2	14	61.07	13.586	41%	28.6%
ISUP 3	61	64.83	17.776	47.3%	50.8%
ISUP 4	31	60.64	23.127	59.1%	80.6%
ISUP 5	27	71.88	46.383	68.8%	85.2%

**Table 3:** The statistical results of the three simulations.

Classification Algorithm	Accuracy Score	Sensitivity	Specificity
Logistic regression	0.8041	0.6163	0.9160
Decision tree classifier	0.6862	0.8490	0.4297
Dense neural network	0.8697	0.8550	0.8223

## Data Availability

The data presented in this study are available on request from the corresponding author.
